# LLM research on public biosignals data is needed to protect patients

**DOI:** 10.1038/s41746-026-02872-3

**Published:** 2026-07-04

**Authors:** James Anibal, Jasmine Gunkel, Hannah Huth, Bradford J. Wood

**Affiliations:** 1 Center for Interventional Oncology, NIH Clinical Center, NIH, Bethesda, MD USA; 2 Department of Engineering Science, Computational Health Informatics Lab, University of Oxford, Oxford, UK; 3 Department of Bioethics, NIH Clinical Center, NIH, Bethesda, MD USA

**Keywords:** Computational biology and bioinformatics, Health care, Mathematics and computing, Medical research

## Abstract

Large language models (LLMs) have expanded the capabilities of AI and created new opportunities in medicine. To promote the development of safe and innovative digital health tools, there must be public biosignals datasets that encourage research and provide benchmarks. Multiple current examples involve data use agreements that restrict LLM studies. This work presents recommendations to ensure that public releases of biosignals data can ethically facilitate LLM research efforts.

## Summary

Publicly available and accessible biosignals datasets are necessary to responsibly develop and evaluate large language models (LLMs) that may be deployed in clinical settings. Expanded data availability may encourage responsible research on the performance and safety of novel AI systems. This could ultimately lead to the implementation of robust LLM tools for biosignals data analysis, better protect patient privacy, and mitigate future risks to the delivery of care.

## Introduction

In recent years, there has been significant progress in the development of AI models for biosignals data. Biosignals include a diverse set of non-invasive modalities that capture the intrinsic behavior of the human body: electrical signals (ECG, EEG), optical measurements such as PPG, recorded voice samples, and wearable sensor data that tracks movement^1,2^. Yet, little work has been published about how large language models (LLMs) might interact with such data, which is uniquely challenging to analyze. Many biosignals can be collected continuously, resulting in noisy datasets with extended steady state periods, and human experts are often unable to reliably interpret these modalities directly (e.g., PPG, many acoustic variables). This has resulted in limited textual context for training LLMs and insufficient benchmarks for the comparison of model results with the performance of human experts. These challenges have introduced ethical uncertainties, particularly in cases where there is vulnerability to sensitive inference. Biosignals are intimate data that have been used to characterize overall wellness, activity, mental state, and daily habits^3,4^. Unlike other types of health data, these non-invasive modalities can be recorded through background processes on wearable or mobile devices, potentially resulting in more frequent capture of private information. For example, ambient scribe applications may record unstructured audio data that contains confidential, identifiable disclosures occurring within conversations between patients and healthcare professionals^5^. The same risks apply to continuous signals from wearable sensors, which may be linked to identifiable metadata like geographic location and have enabled inference of mood changes and measurement of physiological changes during sexual activity^6–8^.

To protect patient privacy, the AI research field must have access to public datasets of biosignals linked to clinical and demographic metadata. These repositories will facilitate a better understanding of how biosignals may be used in proprietary settings by hospitals, insurance companies, government agencies, or any other organization with access to correlated identifiers. For example, in the task of age estimation from PPG, one recent study identified a significant gap between the high level of performance reported on internal Apple datasets and independent experiments conducted on UK Biobank data, which only marginally exceeded the mean prediction baseline^9^.

One core purpose of public biosignals datasets is to support exploratory efforts that may uncover possible adverse impacts in real-world deployment scenarios and promote the development of solutions that protect patients. For example, model training methodologies that do not sufficiently account for class imbalances may cause a downstream decline in performance among underrepresented groups. The MIMIC datasets, available on PhysioNet, have been previously utilized within bias reduction research, potentially lowering the risk of AI errors and exemplifying the value of public data^10–13^. In the imaging domain, public data resources have supported the development and validation of safe diagnostic tools for fields like radiology^14–16^.

Risk mitigation for biosignals is particularly essential, as the AI models developed from such data have the potential to be used by millions of patients well beyond hospital environments. For example, past work has shown that smartwatch data can be used to screen for hypertension and voice AI applications may provide longitudinal insight into Parkinson’s disease progression^17,18^. As medicine continues to prioritize non-invasiveness, standardized resources must be developed for biosignals data to ensure public trust and protect patient rights as LLMs are increasingly integrated into the delivery of healthcare. While limited, there are currently multiple examples of public biosignals datasets that may provide a template for future efforts to create LLM research resources. These include MIMIC-IV-ECG (electrocardiograms), Sleep-EDF database expanded (polysomnography), WESAD (multimodal wearable sensor data), ScientISST MOVE (multimodal movement data), and Bridge2AI-Voice (voice, speech, language)^19–24^.

## Do public biosignals datasets support realistic LLM research?

Multiple existing public biosignals datasets are governed by use agreements (DUAs) that limit LLM research by attempting to restrict the use of application programming interfaces (APIs). This is mainly because the use of such tools may be considered as “sharing the data” with a third party (i.e., technology companies)^25–27^. In these cases, compliant AI research studies must use LLMs that can be run on local devices, limiting research on large or proprietary systems, or privately on specialized cloud platforms that may be inaccessible to many researchers, particularly in resource-constrained settings. The existing guidelines for LLM research are generally vague, require knowledge about the caching, logging, and human review policies of AI companies, and do not clearly specify the allowed platforms.^27^.

This key limitation may challenge the feasibility of academic studies involving models that physicians are already using in clinical contexts. The prevalence of AI use among physicians has doubled between 2023 and 2026^28–30^. Fierce Healthcare, in collaboration with Sermo, surveyed 107 physicians across multiple subspecialties. In this study, the sample population was limited to participants reporting prior use of LLMs for any purpose. Results showed that over 75% of participants informally apply these models in their clinical decision-making processes^29^. This result aligned with a separate cross-sectional study involving 115 healthcare workers in 21 countries, which found that 67% of respondents have used ChatGPT at least once ^30^. Within the cohort of ChatGPT users, applications included clinical practice (46.8%) and research tasks (75.3%)^30^. In a survey of 390 pediatric providers at Boston Children’s Hospital, 50.5% had interacted with an LLM for either administrative work or patient care tasks ^31^. Similarly, LLMs are now widely used as a source of medical information. According to an internal study conducted by OpenAI, ChatGPT receives health questions from over 40 million users per day ^32^. The recent release of ChatGPT Health introduced integrations with b.well, Apple Health, and other wellness applications, allowing model outputs to be grounded in personal data^33^.

Beyond the potential impact on research, use agreements may be difficult to enforce. In a recent example of this, deidentified UK Biobank data from approximately 500,000 participants was briefly listed for sale on Alibaba e-commerce platforms^34^. Following even a single upload to an LLM interface (or API call, depending on retention policies), data may no longer be fully controlled by the institution of origin. Moreover, LLM use may be difficult to confirm unless explicitly stated by the researchers in published work. Evidence from published studies shows that researchers use LLMs for tasks like data analysis, which may not be listed in the methods section of a manuscript^35–37^. ChatGPT alone has over 700 million weekly active users and provides features that support data understanding, organization, summarization, filtering, and modification^38^. Technical barriers that prevent data downloads may limit interactions with LLMs, but also risk constraining many other studies.

If the approval of a data release is contingent upon no downstream user uploading any of the samples into an LLM system, that resource may not be suitable for posting to public repositories. For example, rare disease cohorts may have unique records that are easier to reidentify^39–41^. Datasets from vulnerable populations, where the harms of re-identification may include stigma, discrimination, and deterrence from care, may also be included in this category^42^.

LLMs have introduced a new importance for public data, with specific implications for biosignals. This report presents policy and technical recommendations for academic institutions and hospitals that may develop public data resources, with the aim of ensuring that biosignals are included in LLM research (Table 1). The recommendations in this report are intended for ongoing data generation projects or biosignals datasets that are currently under review for public release.

**Table 1:** Recommendations for facilitating responsible research involving LLMs and public biosignals datasets

Recommendation	Summary
Realistic study design	Clinical biosignals studies should be conducted with the tools that are already being used by stakeholders (i.e., healthcare systems, physicians, and patients). Studies have shown that many practitioners use LLMs like ChatGPT, yet there are significant difficulties in performing LLM research on biosignals data.
Consent processes	Research institutions should ensure adequate informed consent via standardized language for human subject research protections. Potential study participants should be told about how their data may interact with LLMs, including methods, applications, and risks, prior to deciding if they would like to donate biosignals data. Modernized consent systems connected directly to databases are also needed for dynamic, patient-centered consent processes.
Data preparation	Public datasets should be collected, pre-processed, and released with an understanding that there is a high likelihood of downstream contact with LLMs. Processes and methodologies should be developed for anonymization of biosignals (such as standardized tools for voice deidentification). Infrastructure could also be developed for a biosignals data repository that promotes adherence to “FAIR” principles (findable, accessible, interoperable, and reusable)^69^. Examples of open-source resources for data anonymization (non-exhaustive): Microsoft Presidio, LLM-anonymizer, ARX, speaker-anonymization (software package), VoicePAT, pyCANON ^58,70–74^
Researcher protection	Liability assignment should be based on controllable factors. Researchers and institutions should share responsibility, but researchers should not be liable for events like institutional data breaches.

## Recommendation #1: Informed consent processes must explicitly cover LLMs

There is a high likelihood that publicly released information may be captured by data mining efforts, manually uploaded to LLMs, and/or transmitted to model cloud servers via API calls. As such, the best way to prioritize patient rights is through a truly informed consent process. In the current data environment, attempts at downstream regulation are not a substitute for transparent communication at the point of collection. Patients should have control over their digital health records and deserve transparency about how AI tools may interact with their data in the future. Technology companies have faced criticism over opt-out data collection policies that contribute to the “surveillance economy” without explicit permission from the user^43^. Scientific and medical research institutions should not be exempt from the ethical requirement of informed opt-in frameworks, despite concerns about the potential for smaller datasets or selection bias^44^. Past work has found that these risks are frequently overstated and may be addressed through standard statistical methods^45^.

Participants must be made aware of risk factors, including the specific biomarkers that could be inferred from their data, the ways in which this information might be used, the practical irreversibility of model training, the limited control over external AI systems, and the high level of uncertainty about future LLM capabilities. Patients could then choose which technological systems can access their data for AI training or inference purposes. For example, prospective subjects may be willing to donate data for a scoped biomarker study but not an LLM corpus. The consent process would also benefit from digitization, enabling direct linkage to data repositories. Patients could then update or withdraw consent at any time through an online user interface, and their records would be withheld from certain studies or potentially deleted from the dataset.

AI education is also a key aspect of ensuring informed consent within biosignals data collection. Clinical researchers should have the knowledge required to clearly inform patients about risks and address misconceptions about emerging technologies. Current consent interactions often cover the generic future use of data, but do not typically specify AI/LLM risks that might result from patient records being used as input to ChatGPT or a similar platform. Institutions should provide research teams with training resources about LLM applications and risks. Simulation training may help improve how these concepts are communicated to participants with low technical literacy.

## Recommendation #2: Data preparation should be an institutional research focus

Excessive data use restrictions may prevent or disincentivize important research on topics like AI bias and patient safety. Reportedly, only 5% of 519 LLM evaluation studies were based on real patient care data^46^. Given that LLMs are used within search engines, patient education programs, and clinical practice (informally in some cases), the lack of research outputs involving LLMs and biosignals data could ultimately create a harmful knowledge gap^29–34,46–48^. As biosignals become more ubiquitous (e.g., through new wearable technologies or ambient scribes), this data will likely be used in LLM applications. Thorough research with standardized benchmarking is necessary, but this becomes impossible if usage guidelines are too restrictive.

To solve this problem, greater institutional emphasis should be placed on privacy-aware data processing before public release instead of post hoc regulation of LLM research. Algorithms may be designed to automatically remove identifiable and/or sensitive elements from biosignals. In the adjacent domain of computer vision, there have been many research efforts to develop anonymization tools for facial images. Studies involving these methods, which include digital masking, face replacement with synthetic avatars, and selective distortion of facial features, have reported re-identification rates below 5% in some settings^49–51^. Recent advances in deep learning have achieved comparable de-identification results on datasets containing images of public figures (e.g., VGGFace2, CelebA)^52–56^. Similar architectures could be used to modify patient-specific signatures in biosignals data. For example, features of voice recordings, including pitch, cadence, or timbre, could be changed to obscure speaker identity while preserving the ability to extract relevant features required for clinical model development (e.g., jitter measures used in the Dysphonia Severity Index)^57^. Newly developed tools like “LLM-anonymizer”, an open-source framework that uses Llama models to redact identifiable clinical information, may also be adapted for multimodal contexts^58^. In the future, similar methods could be fine-tuned to mask specific elements of speech or wearable data that would facilitate sensitive inference tasks, such as prediction of stigmatized social behaviors.

## Recommendation #3: Researchers should not assume liability for organizational data security

Data security should be considered at the institutional level through risk assessments and regulatory processes prior to public release. Individual researchers bear responsibility for institutional review board (IRB) compliance, transparent reporting, and management of data on local devices, but should not be held liable for breaches on institutional servers or other systemic failings that may affect public data. Undue liability requires researchers to be responsible for circumstances beyond their control, creates a shift in accountability, and introduces vulnerability that may discourage academic innovation. For example, existing access agreements may require the researcher to use “state-of-the-art security measures” or “administrative, technical, and physical safeguards” after downloading the data^59–62^. However, the operative meaning of these concepts is not always clearly defined in the agreements, which may instead reference external standards that have different interpretations across institutions or geographic regions^63–68^. This could create compliance uncertainty for researchers studying or evaluating LLMs. The institutional entities that own the data should be responsible for implementing realistic risk mitigation strategies prior to release, and standardized guidelines for data management should be adopted across institutions to protect researchers.

## Conclusion

LLMs may soon be built into clinical workflows that include biosignals data. To reduce risk and protect patients, careful research must be performed on public datasets prior to deployment in downstream private settings. Inadequate governance structures for biosignals data may discourage innovation, disrespect patient privacy, and obstruct standardized benchmarking experiments. Given the rapid adoption of LLMs, immediate attention to biosignals access and governance is necessary to preserve ethical research processes and improve downstream AI deployments.

## Data Availability

No datasets were generated or analyzed for this study.
